# Nutrition interventions in women with polycystic ovary syndrome: a systematic review

**DOI:** 10.1007/s00394-026-04030-7

**Published:** 2026-06-29

**Authors:** Elif Akbaş, Merve Samancı, Yasemin Ertaş Öztürk

**Affiliations:** 1 Department of Nutrition and Dietetics, Faculty of Health Sciences, Marmara University, Istanbul, Turkey; 2 Department of Nutrition and Dietetics, Faculty of Health Sciences, Avrasya University, Trabzon, Turkey; 3 Department of Nutrition and Dietetics, Faculty of Health Sciences, Ondokuz Mayıs University, Samsun, Turkey

**Keywords:** Polycystic ovary syndrome, Nutrition, Dietary interventions, Calorie-restricted, Intermittent fasting

## Abstract

**Purpose:**

This systematic review aimed to synthesize current evidence on the effects of various dietary interventions on anthropometric, metabolic, hormonal, inflammatory, and oxidative stress parameters in women with polycystic ovary syndrome (PCOS).

**Methods:**

The review followed the PRISMA guidelines and was prospectively registered in PROSPERO (CRD42025641781). Searches were conducted in PubMed, Cochrane Library, EBSCO, Science Direct, Web of Science, National Thesis Center, Google Scholar, and DergiPark Academic for studies published between February 2015 and February 2025. Experimental and observational studies were included if they evaluated the independent effect of dietary interventions in adult women with PCOS. Methodological quality was assessed using the Joanna Briggs Institute (JBI) critical appraisal tools.

**Results:**

A total of 38 studies were included, covering interventions such as calorie-restricted diets, low-glycemic index/load diets, ketogenic diets, intermittent fasting, dietary approaches to stop hypertension, Mediterranean-style, and other diets. Most dietary interventions demonstrated beneficial effects on body weight, body mass index, and waist circumference, as well as improvements in insulin sensitivity, reproductive hormone regulation, and menstrual regularity. However, findings related to lipid metabolism, inflammatory markers, and oxidative stress outcomes were inconsistent.

**Conclusion:**

Current evidence indicates that dietary interventions are crucial in improving the management of metabolic, anthropometric, hormonal, and clinical outcomes in women with PCOS**.** Nevertheless, the heterogeneity of dietary approaches, study designs, and outcome measures highlights the need for long-term randomized controlled trials to establish more conclusive recommendations.

**Supplementary Information:**

The online version contains supplementary material available at 10.1007/s00394-026-04030-7.

## Introduction

Polycystic ovary syndrome (PCOS) is one of the most common endocrine disorders among women of reproductive age, with a global prevalence of approximately 10–15% [1]. Stein and Leventhal first described PCOS in 1935 as a syndrome characterized by hirsutism, chronic anovulation, bilateral ovarian enlargement, and obesity [2]. Although its etiopathogenesis remains complex and not fully elucidated, PCOS is currently diagnosed according to established international criteria, most commonly the Rotterdam criteria, which require the presence of at least two of the following: oligo/anovulation, hyperandrogenism, or polycystic ovarian morphology, after exclusion of related disorders [3, 4].

The incidence of PCOS appears to be increasing in both developing and developed countries as a result of lifestyle changes in diet quality, decreased physical activity, environmental endocrine-disrupting chemicals, changing light exposure, sleep disturbance, increased stress levels, and other environmental factors [5]. Women with PCOS often present with diverse symptoms, including menstrual irregularities, hirsutism, infertility, obesity, acne, and androgenic alopecia, while also facing psychological challenges such as anxiety, depression, and poor body image. Furthermore, PCOS is associated with increased risk of insulin resistance (IR), impaired glucose tolerance, type 2 dDiabetes mellitus (T2DM), hypertension, dyslipidemia, cardiovascular diseases, and hormone-related cancers such as breast and endometrial cancer [6–8].

Nearly 75% of people with PCOS are overweight and/or have obesity, and both people with moderate weight and those with excess weight show central adiposity. Metabolic Syndrome (MetS) in this population is reported to be approximately 43%, primarily driven by IR, hyperinsulinemia, and dyslipidemia, closely linked to central obesity [9]. Ovulatory dysfunction, affecting up to 90% of women with PCOS, is a major contributor to infertility, further compounded by obesity, hyperandrogenemia, and elevated luteinizing hormone (LH) concentrations [10].

Given the challenges of long-term weight management in PCOS, conventional lifestyle interventions may be less effective compared with those in healthy women. This may be caused by PCOS-related hormonal abnormalities such as hyperandrogenemia or IR, leading to irregular energy balance, dietary intake, gut hormone control, or a changed metabolism due to decreased postprandial thermogenesis [15]. A modest 5–10% weight reduction has been associated with improvements in clinical symptoms of PCOS, including ovulation restoration, an increase in pregnancy rates, and decreased insulin and androgen levels [16]. Increasing physical activity combined with dietary modification remains the most widely recommended strategy for women with PCOS who are living with overweight and obesity [11].

Although weight loss is known to improve clinical and metabolic outcomes in women with PCOS, there is still no consensus on the most effective dietary strategy for long-term management. Recent evidence highlights several nutritional approaches, including low-calorie, low-carbohydrate; Dietary Approaches to Stop Hypertension (DASH), Mediterranean, and ketogenic diets, which have been frequently investigated for their potential benefits [12, 13]. These dietary models have demonstrated improvements in IR, fasting glucose, and lipid metabolism, and favorable effects on anthropometric parameters such as body weight and body mass index [13]. In particular, DASH and calorie-restricted diets appear most effective in reducing insulin resistance and abdominal obesity [14]. By contrast, the Mediterranean diet seems to exert additional anti-inflammatory and reproductive benefits, while restrictive regimens such as the ketogenic diet face challenges of long-term adherence [12].

Currently, no universal treatment protocol exists for PCOS, and pharmacological therapies are primarily symptomatic. No medications are specifically approved for PCOS, so management strategies should be individualized and tailored to patients’ clinical needs. While pharmacological interventions may provide short-term symptom relief in acute phases, long-term complications are more effectively addressed through sustainable lifestyle modifications, particularly diet, physical activity, and behavioral strategies [15–18]. International evidence-based guidelines emphasize lifestyle modification as the first-line approach to improve insulin resistance, metabolic health, and reproductive outcomes in women with PCOS [19]. Therefore, the primary objective of this systematic review was to evaluate the available scientific evidence on the effects of diverse dietary interventions on anthropometric, metabolic, hormonal, inflammatory, and clinical outcomes in women with PCOS.

## Methods

This systematic review was conducted following the Preferred Reporting Items for Systematic Reviews and Meta-Analysis (PRISMA) guidelines (Supplementary File 1) [20]. We registered the protocol in the International Prospective Register of Systematic Reviews (PROSPERO), registration number CRD42025641781.

### Data sources and search strategy

A comprehensive systematic literature search was performed to identify studies investigating the effects of nutrition interventions on metabolic, anthropometric, inflammatory, oxidative stress, and hormonal parameters in patients with PCOS. Searches were conducted using PubMed, the Cochrane Library, EBSCO, Science Direct, Web of Science, National Thesis Center, Google Scholar, and DergiPark Academic search engines. Search strategies included the following terms (‘‘polycystic ovary syndrome’’ OR ‘‘PCOS’’ OR ‘‘polycystic ovarian syndrome’’ OR ‘‘polycystic ovary disease’’ OR ‘‘ovarian cysts’’ OR ‘‘Stein-Leventhal syndrome’’ OR ‘‘Stein Leventhal syndrome’’) AND (‘‘diet*’’ OR ‘‘nutrition*’’) that were applied in both English and Turkish. Detailed search strategies for each database are provided in Supplementary File 2. The search was restricted to articles published between February 2015 and February 2025. The search for this study was carried out independently by two researchers (EA and MS) from 15 February to 05 March 2025.

### Inclusion and exclusion criteria

Research questions were defined a priori using the PICOS (Population, Intervention, Comparison, Outcomes, and Study design) framework. The inclusion and exclusion criteria for the search are summarized in Table 1.

**Table 1:** Summary of the PICOS criteria used to identify studies to be included

Parameter	Description
Population	Women of reproductive age/adult with polycystic ovary syndrome (PCOS) based on the NIH (1990), the Rotterdam ESHRE/ASRM (2003), or the AE-PCOS Criteria (2006) and trials where a general practitioner or specialist clinician had verified the PCOS diagnosis
Intervention	Calorie-restricted diet (CRD), low-carbohydrate diet, high-protein diet (HPD), low-fat diet, Dietary Approaches to Stop Hypertension (DASH) diet, low-glycemic index diet (LGID) and/or low-glycemic load diet (LGLD), vegetarian/vegan diet, Mediterranean diet (MD), ketogenic diet (KD), intermittent fasting (IF), very-low-calorie diet (VLCD), or other dietary interventions
Comparison	Usual diet or no intervention or diet or medical treatment or pre-situation
Outcomes	Blood Tests: Glucose and Insulin Metabolism: Homeostatic Model Assessment of Insulin Resistance (HOMA-IR), Fasting Insulin (FI), Fasting Glucose (FG), Glycated Hemoglobin (HbA1c), Total Cholesterol (TC), Low-Density Lipoprotein Cholesterol (LDL-C), High-Density Lipoprotein Cholesterol (HDL-C), Triglycerides (TG), Apolipoprotein A (APOA), Very-Low-Density Lipoprotein Cholesterol (VLDL-C), Total Testosterone (TT), Free Testosterone (FT), Free Androgen Index (FAI), Sex Hormone-Binding Globulin (SHBG), Dehydroepiandrosterone Sulfate (DHEAS), Luteinizing Hormone (LH), Follicle-Stimulating Hormone (FSH), LH/FSH ratio, Estradiol (E2), Progesterone, Anti-Müllerian Hormone (AMH), Insulin-like Growth Factor-1 (IGF-1), Prolactin, Thyroid-Stimulating Hormone (TSH), High-Sensitivity C-Reactive Protein (hs-CRP), C-Reactive Protein (CRP), Tumor Necrosis Factor-alpha (TNF-α), Calprotectin, Malondialdehyde (MDA), Nitric Oxide (NO), Total Antioxidant Status (TAS), Glutathione (GSH), Alanine Aminotransferase (ALT), Aspartate Aminotransferase (AST), Systolic Blood Pressure (SBP), Diastolic Blood Pressure (DBP), 25-hydroxy vitamin D (25-OH D), 2,2-diphenyl-1-picrylhydrazyl (DPPH) radical scavenging activity Anthropometric Measurements: Body Weight, Waist Circumference (WC), Body Mass Index (BMI), Body Fat Mass (BFM), Body Fat Percentage (BFP), Fat-Free Mass (FFM), Lean Body Mass (LBM), Lean Muscle Mass (LMM), Muscle Mass (MM), Body Water (BW), Hip Circumference (HC), Waist-to-Hip Ratio (WHR), Mid-Upper Arm Circumference (MUAC), Chest Circumference, Neck Circumference, Visceral Adipose Tissue (VAT), Basal Metabolic Rate (BMR) Reproductive and Clinical Outcomes: Menstrual cycle length, frequency, and regularity, Ovulatory cycles, Oocyte retrieval, Implantation rate, Pregnancy outcomes (clinical pregnancy, ongoing pregnancy, live birth, miscarriage); Hyperandrogenism-related symptoms (Hirsutism assessed by Ferriman–Gallwey Score, Acne, Alopecia/hair loss)
Study design	Experimental studies (randomized controlled trial, quasi-experimental studies, pre-post intervention study, pilot study) or observational studies (cross-sectional study, case–control study, cohort study)

The exclusion criteria adopted in the present study are as follows: (1) meta-analyses, systematic reviews, literature reviews, narrative reviews, letters to the editor, conference abstracts, study protocols, and case reports; (2) full text was unavailable; (3) studies with unclear data or languages other than English or Turkish; (4) sample size fewer than 10 participants or an intervention duration shorter than 4 weeks; (5) studies that included combined interventions such as exercise, supplementation, or other lifestyle modifications, in which the specific effect of the dietary intervention could not be assessed independently; and (6) nonhuman, preclinical, and animal studies.

### Characteristics of dietary ınterventions

The characteristics of the dietary intervention models included in this review are summarized in Table 2. These definitions were based on commonly accepted descriptions in the literature and reflect the macronutrient composition or structural features of each dietary model.

**Table 2:** Definitions of dietary intervention models

Dietary model	Definition
Ketogenic diet (KD)	High-fat, adequate-protein, very low-carbohydrate dietary pattern (typically < 50 g/day carbohydrate) designed to induce nutritional ketosis. Variations include classical KD, medium-chain triglyceride (MCT) diet, modified Atkins diet (MAD), and low-glycemic-index treatment approaches
DASH diet	Dietary pattern emphasizing fruits, vegetables, whole grains, lean protein, and low-fat dairy products, with reduced sodium intake (typically ≤ 1500–2300 mg/day) and limited processed foods
Low-glycemic-ındex diet (LGID)	Diet with standard macronutrient distribution (~ 50% carbohydrate, ~ 30% fat, ~ 20% protein) prioritizing carbohydrate sources with a low glycemic index to moderate postprandial glucose response
Calorie-restricted diet (CRD)	Energy intake is reduced by approximately 500–1000 kcal/day below estimated requirements, typically composed of ≤ 60% carbohydrate, ≤ 30% fat, and the remainder from protein
Intermittent fasting (IF)	Time-restricted feeding pattern (e.g., 16:8 model) involving defined fasting periods; may or may not include concurrent caloric restriction. Macronutrient composition generally follows balanced dietary recommendations
Mediterranean diet (MD)	Plant-based dietary pattern rich in fruits, vegetables, legumes, whole grains, nuts, seeds, and olive oil; moderate intake of fish, dairy, and poultry; limited red meat consumption
Vegetarian diet	Dietary pattern excluding meat; may include dairy and/or eggs depending on subtype (lacto-, ovo-, lacto-ovo-, or pescatarian)
Vegan diet	Dietary pattern excluding all animal-derived products
Low-carbohydrate diet	Dietary pattern reducing total carbohydrate intake, typically below 45% of total energy intake; no universally standardized carbohydrate threshold
High-protein diet (HPD)	A dietary pattern in which protein provides more than 20% of total energy intake; weight-loss protocols often target ~ 30% of total energy from protein
Low-fat diet	A dietary pattern in which total fat intake is reduced below 30% of total energy intake, often lower in clinical interventions

### Study selection and selection criteria

Two reviewers (EA and MS) independently screened titles and abstracts for eligibility. Full-text screening was conducted to identify studies meeting the inclusion criteria (EA and MS). The authors determined an order of reasons for exclusion that was discussed prospectively. Studies excluded during the full-text screening were classified according to the specific reason for exclusion. In cases of differences or disagreements between the two reviewers, a third reviewer (YEO) was consulted to reach a consensus.

### Data extraction

The following data were extracted by two reviewers (EA and MS) using a standardized protocol: 1) author’s name, year, country; 2) participants’ characteristics, including the sample size of participants who completed each of the intervention and control groups, age, BMI, and criteria used to define PCOS; 3) study design; 4) study duration; 5) dietary characteristics in the intervention and control groups; and 6) the direction of changes in major findings related to clinical, hormonal, inflammatory, and metabolic outcomes of interest that were reported between the intervention and control groups or in a pre-post situation in each study. For any missing or unclear information, data were recorded as not reported.

### Quality assessment

The quality of the included studies was independently assessed by two of the authors (EA and MS) using the randomized controlled trial (13 items); the quasi-experimental studies (9 items); and the cohort studies (11 items) checklists published by the Joanna Briggs Institute (JBI). The quality of the studies was classified into three types: poor, moderate, and good, according to the assessment results. The methodological quality level of the studies included in the research was considered ‘‘poor’’ if less than 50% of the items were evaluated as ‘‘yes’’, ‘‘moderate quality’’ if 51–80% of the checklist items were assessed as ‘‘yes’’, and ‘‘"good quality’’" if more than 80% of the items were evaluated as ‘‘"yes’’" [21].

## Results

### Study selection and characteristics

**Fig. 1:**
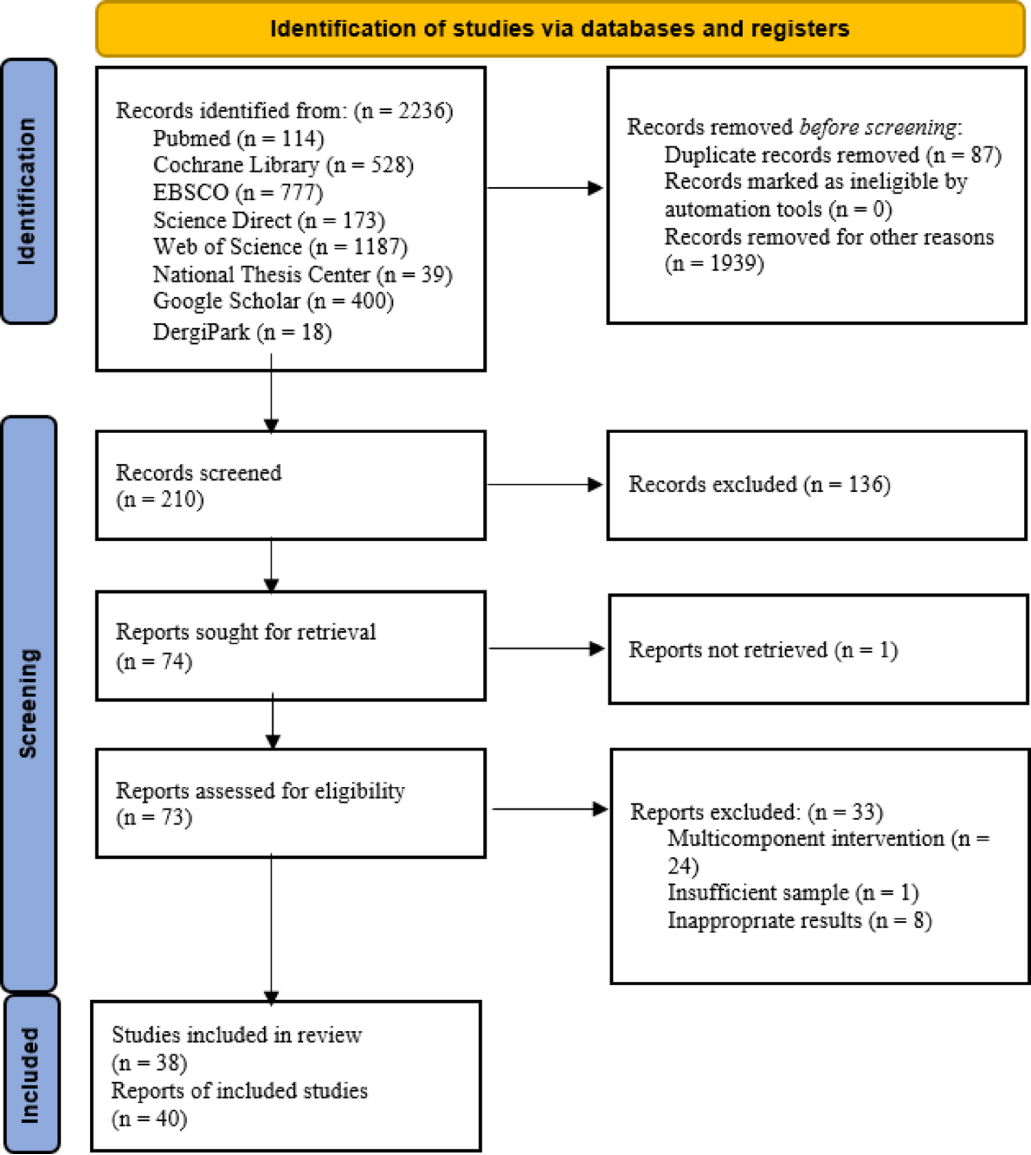
Prisma flow chart

The PRISMA flow chart for the selection phase and systematic review of the studies is given in Fig. 1. A total of 2236 records were retrieved from the databases analyzed. After removing duplicates and irrelevant records, 210 studies were screened based on their titles and abstracts, of which 136 were excluded due to unrelated topics. One full-text article could not be retrieved [22]. Among the 73 studies whose full text was analyzed, 24 were excluded due to multicomponent interventions where the effect of nutrition could not be isolated, one was excluded due to insufficient sample size (< 10 participants), and eight were excluded due to reporting inappropriate or irrelevant outcomes. Thirty-eight studies, reported across 40 different sources, were included in the systematic review.

Table 6 summarizes the characteristics and key findings of the included studies. These studies were conducted in 13 countries across three continents, with the majority originating from Asia (23 studies, 57.5%) [23–44], followed by Europe (8 studies, 20%) [45–52] and America (9 studies, 22.5%) [53–61].

The included studies addressed various dietary interventions, including KD [39, 42, 53], the DASH diet [24, 26, 29], LGID [40, 41, 45, 46, 54–56], CRD [25, 31, 34, 37, 47–50, 57, 62], IF [23, 27, 28, 30, 32, 43], and other approaches such as high-protein, low-AGE, Mediterranean-style, pulse-based, and low-starch/low-dairy diets [33, 35, 36, 38, 44, 51, 52, 58–61].

### Quality evaluation

Quality assessment results are presented for randomized controlled trials in Table 3, intervention studies in Table 4, and cohort studies in Table 5. The majority of the randomized controlled trials were rated as moderate quality, while only three studies [29, 58–60] achieved a good quality rating. Most of the quasi-experimental studies were classified as good quality, except three studies, [35, 46, 62], that were rated as moderate quality. Regarding cohort studies, the study by Yang et al. [42] was rated as moderate, whereas Kataoka et al. [51] was assessed as having good quality.

**Table 3:** Critical evaluation checklist for JBI randomized controlled trials

	Critical evaluation checklist questions for JBI randomized controlled trials													Quality level of the study (%)
Study, year	Q1	Q2	Q3	Q4	Q5	Q6	Q7	Q8	Q9	Q10	Q11	Q12	Q13	
Asemi et al., 2015 [24]	Y	U	Y	Y	Y	Y	U	Y	Y	Y	N	Y	Y	76.9/ Moderate
Azadi-Yazdi et al., 2017 [26]	Y	U	Y	Y	U	Y	U	Y	Y	Y	N	Y	Y	69.2/ Moderate
Foroozanfard et al., 2017 [29]	Y	Y	Y	Y	N	Y	U	Y	Y	Y	Y	Y	Y	84.6/ Good
Becker et al., 2015 [54]	Y	U	Y	N	N	Y	U	Y	Y	Y	N	Y	Y	61.5/ Moderate
Hoover et al., 2021 [55]	Y	U	Y	Y	U	Y	U	Y	Y	N	U	Y	Y	61.5/ Moderate
Łagowska et al., 2022 [45]	U	U	Y	N	N	Y	U	Y	Y	Y	N	Y	Y	53.8/ Moderate
Sordia-Hernández et al., 2015 [56]	U	U	Y	U	U	Y	U	Y	Y	Y	N	Y	Y	53.8/ Moderate
Swora-Cwynar et al., 2016 [47]	U	N	Y	N	N	Y	U	Y	Y	Y	Y	Y	Y	61.5/ Moderate
Le Donne et al., 2019 [48]	U	U	Y	Y	N	Y	U	Y	Y	Y	Y	Y	Y	69.2/ Moderate
Kulshreshtha et al., 2024 [31]	Y	U	Y	Y	N	Y	U	Y	Y	Y	Y	Y	Y	76.9/ Moderate
Nybacka et al., 2017, 2019 [49, 50]	Y	U	Y	N	N	Y	U	Y	Y	Y	Y	Y	Y	69.2/ Moderate
Cezlan and Sevim, 2024 [27]	Y	U	Y	U	N	Y	N	Y	Y	Y	N	Y	Y	61.5/ Moderate
Jiang et al., 2025 [30]	Y	U	Y	N	N	Y	U	Y	Y	Y	Y	Y	Y	69.2/ Moderate
Abu Salma et al., 2024 [23]	Y	U	Y	U	U	Y	U	Y	Y	Y	Y	Y	Y	69.2/ Moderate
Colonetti et al., 2025 [58]	Y	Y	Y	U	Y	Y	Y	Y	Y	Y	N	Y	Y	84.6/ Good
Karamali et al., 2018 [44]	Y	Y	Y	N	N	Y	U	Y	Y	Y	Y	Y	Y	76.9/ Moderate
Kazemi et al., 2018, 2020 [59, 60]	Y	Y	Y	N	Y	Y	Y	Y	Y	Y	Y	Y	Y	92.3/ Good
Mei et al., 2022 [33]	Y	U	Y	U	U	Y	U	Y	Y	Y	N	Y	Y	61.5/ Moderate
Ozdemir et al., 2025 [36]	Y	U	Y	U	U	Y	U	Y	Y	Y	N	Y	Y	61.5/ Moderate
Papakonstantinou et al., 2016 [52]	Y	U	N	N	N	Y	U	Y	Y	Y	N	Y	Y	53.8/ Moderate
Sanlı Ak et al., 2017 [38]	N	U	U	N	N	Y	N	Y	Y	Y	Y	Y	Y	53.8/ Moderate
Y: Yes, N: No, U: Unclear, Q: Question

**Table 4:** Critical evaluation checklist for JBI quasi-experimental studies

	Critical evaluation checklist for JBI quasi-experimental studies									Quality level of the study (%)
Study, year	Q1	Q2	Q3	Q4	Q5	Q6	Q7	Q8	Q9	
Seven Avuk et al., 2021 [39]	Y	N	Y	Y	Y	Y	Y	Y	Y	88.9/ Good
Palafox-Góomez et al., 2023 [53]	Y	N	Y	Y	Y	Y	Y	Y	Y	88.9/ Good
Shishehgar et al., 2019 [40]	Y	Y	Y	Y	Y	Y	Y	Y	Y	100.0/ Good
Shishehgar et al., 2023 [41]	Y	Y	Y	Y	Y	Y	Y	U	Y	88.9/ Good
Szczuko et al. 2019 [46]	Y	Y	N	Y	Y	Y	Y	N	Y	77.8/ Moderate
Ataç Asan et al., 2017 [25]	Y	Y	Y	Y	Y	Y	Y	Y	Y	100.0/ Good
Mahmoud 2018 [62]	Y	N	Y	Y	Y	Y	Y	U	Y	77.8/ Moderate
Moini et al., 2019 [34]	Y	N	Y	Y	Y	Y	Y	Y	Y	88.9/ Good
Soares et al., 2016 [57]	Y	N	Y	Y	Y	Y	Y	Y	Y	88.9/ Good
Özturan Sirin et al., 2020 [37]	Y	N	Y	Y	Y	Y	Y	Y	Y	88.9/ Good
Feyzioglu et al., 2023 [28]	Y	N	Y	Y	Y	Y	Y	Y	Y	88.9/ Good
Li et al., 2021 [32]	Y	N	Y	Y	Y	Y	Y	Y	Y	88.9/ Good
Zangeneh et al., 2015 [43]	Y	Y	Y	Y	Y	Y	Y	Y	Y	100.0/ Good
Naji et al., 2024 [35]	Y	N	Y	Y	Y	Y	Y	U	Y	77.8/ Moderate
Phy et al., 2015 [61]	Y	N	Y	Y	Y	Y	Y	Y	Y	88.9/ Good
Y: Yes, N: No, U: Unclear, Q: Question

**Table 5:** Critical evaluation checklist for JBI cohort studies

	Critical evaluation checklist for JBI cohort studies											Quality level of the study (%)
Study, year	Q1	Q2	Q3	Q4	Q5	Q6	Q7	Q8	Q9	Q10	Q11	
Yang et al., 2022 [42]	Y	Y	Y	U	N	Y	Y	Y	Y	N	Y	72.7/ Moderate
Kataoka et al., 2019 [51]	Y	Y	Y	Y	Y	Y	Y	Y	N	N	Y	81.8/ Good

Y: Yes, N: No, U: Unclear, Q: Question

### Relationship between dietary ınterventions and anthropometric measurements

In more than thirty studies, dietary interventions were reported to result in significant reductions in body weight and BMI among women with PCOS. These improvements were observed following interventions involving KD [39, 42, 53], DASH [26, 29], LGID [40, 41, 45, 54], CRD [23, 25, 27, 36, 37, 47, 48, 57], IF [23, 27, 32], HPD [38], VLCD [51], ad libitum low-starch/low dairy diet [61], and MD with low-carbohydrate or low-fat variations [33]. Additionally, some studies reported reductions in BMI only [24, 28, 30, 35, 50] while others observed reductions solely in body weight [31, 34, 62].

Among circumference measurements, WC was significantly reduced in more than twenty studies. These reductions were reported in interventions using KD [39, 53], DASH [24, 26], LGID [40, 41], CRD [25, 27, 34, 37, 47–50], IF [27, 30], and other dietary interventions including Mediterranean diet combined with a low-carbohydrate diet [33], a very-low-calorie diet (VLCD) [51], a six-meal isocaloric pattern [52], and an ad libitum low-starch/low-dairy diet [61]. Reductions in HC [24–27, 30, 37–39, 48, 51, 52, 54] and WHR were also commonly reported [25, 28, 30, 33, 36, 37, 39, 41, 48, 51, 54, 61]. Additionally, significant decreases in NC [27, 37–39], waist-to-height ratio [37–39], chest circumference [39], and fat/lean ratio [48] were observed in several studies. Also, MUAC was significantly reduced in studies implementing KD [36], CRD, and IF [27].

Regarding body composition, significant reductions were reported in BFP [23, 27, 32, 33, 36–39, 45, 47, 48, 50, 54, 61], BFM [23, 25–27, 32, 38, 39, 42, 47, 48], LBM [25, 26, 38, 42, 47, 48, 50], LMM [42], FFM [23, 27, 38, 39], VAT [23, 27, 32, 36, 38, 39, 42], MM [23, 39], and BW [27, 38, 39]. However, one six-month CRD study reported an increase in BW [48]. In addition, reductions in arm fat, leg fat, and trunk fat were reported in [27, 39], and one study applying an energy-restricted low-AGE or standard AGE diet showed a decrease in trunk fat percentage [36]. As for BMR, three studies reported significant decreases following the dietary intervention [27, 39, 42] (Table 6).

**Table 6:** Characteristics and major findings of the included studies on the effect of nutrition interventions in patients with PCOS

Author, year (reference), Country	Participants’ characteristics and PCOS definitions	Study design	Duration	Intervention	Control	Dietary Adherence Monitoring	Major findings
*Ketogenic Diets*							
Seven Avuk et al., 2021 Türkiye [39]	n = 13 Age: 18–44 years BMI: ≥ 25 kg/m^2^ PCOS definition: Rotterdam criteria	Quasi-experimental	4 weeks	KD: 500 kcal reduction from the total daily energy requirement, including carbohydrates at < 20 g/day, protein at 0.8–1.2 g/kg/day, and daily fat intake > 30–40 g/day	None	Adherence was monitored through four follow-up visits (every 7–10 days); diet/PA/body composition monitored	↓ Body weight, BMI, BMR, BFP, BFM, FFM, MM, BW, VAT ↓ Arm fat (R/L), leg fat (R/L), trunk fat ↓ Arm muscle (R/L), leg muscle (R/L), trunk muscle ↓ WC, HC, WHR, waist/height ratio, MUAC, chest, and neck circumference ↓ FG, HDL, FI, HOMA-IR, prolactin, IGF-1 ↑ SHBG, TNF-α
Palafox-Gomez et al., 2023, Mexico [53]	n = 12 Age: 22–40 years BMI: Overweight/ obese PCOS definition: Rotterdam criteria (IR (HOMA-IR > 1.96), previous failed IVF cycle)	Quasi-experimental	14 ± 11 weeks	KD: 1800–2000 cal/day, consisting of daily consumption of ≤ 50 g of total carbohydrates (around 15%), 1.5 g/kg/day protein (25%), and the remainder with fat (≥ 60%)	None	Self-logged dietary intake; records reported to the nutritionist	↓ Body weight, BMI, WC ↓ TG, FG, FI, HOMA-IR ↑ Cycle implantation rate, clinical pregnancy, live birth rate, ongoing pregnancy/live birth rate
Yang et al., 2022 China [42]	n = 55 Age: 20–40 years BMI: ≥ 24 kg/m^2^ PCOS definition: Rotterdam criteria and Chinese guidelines (Hyperuricaemia and non-hyperuricaemia group)	Prospective cohort study	12 weeks	KD: Daily consumption of ≤ 50 g of total carbohydrates (5–10%), 70–75% fat, and 18–27% protein, energy based on measured basal metabolism (via indirect calorimetry or bioelectrical impedance)	None	Daily counselling sessions and question-and-answer meetings	↓ Body weight, BMI, BMR, BFM, LMM, LBM, VAT ↓ TG, FG, ALT, AST ↑ LDL-C, TC, uric acid (non-hyperuricaemia group) ↓ APOA (non-hyperuricaemia group)
*DASH Diet*							
Asemi et al., 2015 Iran [24]	n = 48 (I: 24; C:24) Age: 18–40 years BMI: ≥ 25 kg/m^2^ PCOS definition: Rotterdam criteria	RCT	8 weeks	DASH: 350–700 kcal reduction from the total daily energy requirement, consisting of 52% carbohydrates, 18% proteins, and 30% total fats. The sodium intake was less than 2400 mg/day	The diet, specifically designed based on the traditional Iranian dietary pattern, contained 52% carbohydrates, 18% protein, and 30% total fat	NR	Significant improvements were observed in the DASH group compared to the control group ↓ BMI, WC, HC ↓ FI, HOMA-IR, hs-CRP,
Azadi-Yazdi et al., 2017 Iran [26]	n = 55 (I: 27; C:28) Age: 20–40 years BMI: 25–40 kg/m^2^ PCOS definition: Rotterdam criteria	RCT	12 weeks	DASH: 350–500 kcal reduction from the total daily energy requirement, consisting of 50–55% carbohydrates, 15–20% protein, and 25–30% total fats. Rich in fruits, vegetables, whole grains, low-fat dairy low in saturated fats, cholesterol, refined grains, sweets, and sodium < 2400 mg/day	Based on national healthy eating guidelines, 350–500 kcal/day energy reduction; 50–55% carbohydrates, 15–20% protein, 25–30% fat	Monthly 3-day food records plus monthly dietitian-administered 24-h dietary recalls and bimonthly dietitian visits	Significant improvements were observed in the DASH group compared to the control group: ↓ Body weight, BMI, BFM ↓ Androstenedione, FAI ↑ SHBG, DPPH Although no significant between-group differences were observed, both the DASH and control diet groups demonstrated within-group reductions in: ↓ WC, HC, LBM
Foroozanfard et al., 2017 Iran [29]	n = 60 (I: 30; C:30) Age: 18–40 years BMI: > 25 kg/m^2^ PCOS definition: Rotterdam criteria	RCT	12 weeks	DASH: 350–700 kcal reduction from the total daily energy requirement, consisting of 52–55% carbohydrates, 16–18% proteins, and 30% total fats. The diet is rich in fruits, vegetables, whole grains, and low-fat dairy; it is low in saturated fats, cholesterol, refined grains, and sweets, with sodium intake limited to < 2400 mg/day. Individualized menus and exchange lists are provided	The diet, specifically designed based on the traditional Iranian dietary pattern, contained 52–55% carbohydrates, 16–18% protein, and 30% total fat	Three-day dietary records and physical activity records at baseline and weeks 3, 6, 9, and 12	Significant improvements were observed in the DASH group compared to the control group: ↓ Body weight, BMI ↓ FI, HOMA-IR, HOMA-B, MDA ↓ AMH, FAI, ↑ QUICKI, NO ↑ SHBG
Low-Glycemic-Index Diets							
Becker et al., 2015 Brazil [54]	n = 26 (I: 14; C:12) Age: 18–40 years BMI: 25–40 kg/m^2^ PCOS definition: NR (Women diagnosed with infertility)	RCT	12 weeks	LGID: Diet providing 20 kcal/kg/day; ~ 50% carbohydrates, 20% protein, 30% fat; moderate-to-high fiber content; individualized portions with household measures, olive oil (5–10 mL/day), and dried fruits provided	Participants maintained their usual diet	Three-day food records at baseline, week 6, and week 12	Significant improvements were observed in the LGID group compared to the control group ↓ Body weight, BMI, BFP, HC, WHR, ↓ Leptin ↑ Oocyte retrieval Pregnancy rate: 21.4% (3/14)
Hoover et al., 2021 USA [55]	n = 27 Age: 21–50 years BMI: ≤ 45 kg/m^2^ PCOS definition: NIH 1990	Randomized crossover trial	8 weeks per arm (total 20 weeks, including a 4-week washout)	LGLD: 41% CHO, 19% protein, 40% fat; GI ≈50; energy adjusted to maintain body weight, foods fully provided	HGLD: 55% CHO, 18% protein, 27% fat; GI ≈60; isocaloric and foods fully provided	NR	Significant improvements were observed in the LGLD compared to the HGLD**:** ↓ Ghrelin (at 240 min) ↑ Glucagon (at 0, 60, 120, 180 min)
Łagowska et al., 2022 Poland [45]	n = 40 (I1: 19; C:21) Age: 18–45 years BMI: Overweight/ obese PCOS definition: Rotterdam criteria	RCT	20 weeks	LGID: 600 kcal/day deficit (~ 0.5 kg weight loss/week): prepared as five meals/day, consisting of 50% carbohydrates (low–medium GI), 15–20% protein, 25–30% fat. Participants received probiotic supplements, 2 capsules/day providing 12 billion CFU (Lactobacillus rhamnosus)	*The intervention group received the same hypocaloric low-GI diet (identical in energy and macronutrient composition)	Scheduled telephone follow-up contacts (weeks 2, 6, 10, 14, and 18)	↓ Body weight, BMI, BFP ↓ Acetic acid, butyric acid ↓ TC, LDL-C, TG ↑ HDL-C
Shishehgar et al., 2019 Iran [40]	n = 62 (28 PCOS/ 34 eumenorrheic non-hirsute controls) Age: 18–40 years BMI: Overweight/ obese PCOS definition: Rotterdam criteria	Quasi-experimental	24 weeks	LGID: 500 kcal reduction from the total daily energy requirement, consisting of 50% carbohydrates, 20% proteins, and 30% total fats	None	Three-day food records completed twice monthly	In the PCOS group: ↓ Body weight, BMI, WC ↓ DBP, FI, HOMA-IR ↓ TT, FAI ↑ SHBG ↓ Menstrual cycle length ↑ Menstrual frequency and regularity ↓ Hirsutism ↓ Acne prevalence and severity
Shishehgar et al., 2023 Iran [41]	n = 216 (105 PCOS/ 111 eumenorrheic non-hirsute controls) Age: 18–40 years BMI: Overweight/ obese PCOS definition: Rotterdam criteria	Quasi-experimental	24 weeks	LGID: 500 kcal reduction from the total daily energy requirement, consisting of 50% carbohydrates, 20% proteins, and 30% total fats	None	Biweekly three-day food records; adherence defined according to the percentage of total energy derived from carbohydrates, protein, and fat	In the PCOS group: ↓ Weight, BMI, WC, WHR
Sordia-Hernández et al., 2015 Mexico [56]	n = 37 (I: 19; C:18) Age: 18–40 years BMI: 25–40 kg/m^2^ PCOS definition: Rotterdam criteria (Women diagnosed with infertility and anovulation)	RCT	3 months	LGID: 1,200 to 1,500 kcal per day with 45–50% complex carbohydrates, 30–40% fat (including 10–15% monounsaturated, < 10% polyunsaturated, and < 10% saturated fats), 15–20% protein, 20-35 g fiber, a source of omega-3, and foods with a glycemic index below 45	The control diet had the same energy and macronutrient composition as the intervention diet, but was composed of foods with a normal glycemic index (GI between 50 and 75)	Food diary plus monthly clinic visits	Significant improvements were observed in the LGID group compared to the control group: ↑ Ovulatory cycles
Szczuko et al. 2019 Poland [46]	n = 24 Age: NR BMI: 29.68 ± 6.48 kg/m^2^ PCOS definition: Rotterdam criteria	Quasi-experimental	3 months	LGID: 600 kcal reduction from the total daily energy requirement, consisting of approximately 50% carbohydrates (low glycemic index), up to 20% protein (animal and vegetable protein in a 1:1 ratio), and up to 30% fat	None	Dietary interviews and food diaries at two control visits; noncompliance resulted in exclusion from further analysis	↑ Uric acid, glutathione peroxidase
*Calorie-Restricted Diets*							
Ataç Asan et al., 2017 Turkiye [25]	n = 45 (I_1_: 15; I_2_:15; I_3_:15) Age: 20–35 years BMI: 25–35 kg/m^2^ PCOS definition: NR	Quasi-experimental	8 weeks	*CRD: 500 kcal reduction from the total daily energy requirement, consisting of 45–60% carbohydrates, 12–15% proteins, and 25–30% total fats. One group received only dietary intervention, while other groups received either curcumin or white bean extract in addition to dietary intervention	None	Three-day food records were used to assess dietary adherence	↓ Body weight, BMI, WC, HC, WHR, BFM, LBM ↓ DHEAS, LH ↑SHBG
Kulshreshtha et al., 2024 India [31]	n = 134 (I:66; C:68) Age: NR BMI: NR PCOS definition: Rotterdam criteria	RCT	1 year	CRD: 500 kcal reduction from the total daily energy requirement, consisting of 60% carbohydrates, 15% proteins, and 25% total fats. Participants were encouraged to engage in regular physical activity of 20 min of walking at least 3 days a week	Isocaloric diet: Calories being consumed by the subject before intervention. Participants were encouraged to do regular physical activity of 20 min of walking at least 3 days a week	Follow-up visits at 3, 6, and 12 months; two-day dietary recalls and verbal reinforcement of compliance	Significant improvements were observed in the CRD group compared to the control group: ↓ Body weight (0–3 months)
Le Donne et al., 2019 Italy [48]	n = 43 (I_1_: 21; I_2_:10; I_3_:12) Age: 16–45 years BMI: ≥ 25 kg/m^2^ PCOS definition: Rotterdam criteria	RCT	6 months	*CRD: A 1200 kcal diet was administered to all three groups, consisting of 25% fats, 15–18% proteins, and the remaining portion consisting of carbohydrates primarily as low glycemic index foods and a recommendation for primarily low glycemic index foods	The intervention diet had the same energy and macronutrient composition, and in addition, group 2 received myo-inositol and folic acid, whereas group 3 received myo-inositol, D-chiro-inositol, and folic acid	NR	↓ Body weight, BMI, WC, HC, WHR, BFP, BFM, LBM, LBM%, fat/lean ratio ↑ BW ↓ Hirsutism ↓ Oligomenorrhea
Mahmoud 2018, Egypt [62]	n = 30 Age: 20–30 years BMI: 30–34,9 kg/m^2^ PCOS definition: Rotterdam criteria	Quasi-experimental	12 weeks	CRD: 500–1000 kcal reduction from the total daily energy requirement, consisting of 55% carbohydrates, 15% proteins, and 30% total fats	None	NR	↓ Body weight ↓ CRP, LH/FSH ↓ Hirsutism, acne
Moini et al., 2019 Iran [34]	n = 90 Age: 18–40 years BMI: ≥ 28 kg/m^2^ PCOS definition: Rotterdam criteria	Quasi-experimental	12 weeks	CRD: The total daily caloric intake was reduced by at least 1000 kcal/d compared to the pre-intervention intake of 55–60% carbohydrates, 25–30% fat (10% saturated), and 10–15% proteins	None	Self-reported dietary intake for three consecutive days before and after the intervention	↓ Body weight, WC ↓ FI, free testosterone ↓ AMH (only women who had improved menstrual cyclicity)
Nybacka et al., 2017 and 2019, Sweden [49, 50]	n = 43 (I_1_: 14; I2:17; I_3_:12) Age: 18–40 years BMI: > 27 kg/m^2^ PCOS definition: Rotterdam criteria	RCT	4 months	CRD: ≥ 600 kcal reduction compared with pre-intervention conditions, consisting of 55–60% carbohydrates, 25–30% fat (10% saturated), and 10–15% proteins	The other two groups received either exercise alone or exercise combined with dietary intervention	Four-day food records (three weekdays and one weekend day) were collected before and after the intervention	↓ BMI, WC, BFP, LBM ↓ FI, FG, HOMA index, TC, LDL-C ↑ IGF-1 ↓ Free testosterone
Ozturan Sirin et al., 2020 Turkiye [37]	n = 48 Age: 18–45 years BMI: ≥ 25- < 40 kg/m^2^ PCOS definition: NR	Quasi-experimental	8 weeks	CRD: 500–1000 kcal reduction from the total daily energy requirement, consisting of 50–55% carbohydrates, 15–20% proteins, and 25–30% total fats (< 10% saturated fat)	None	Baseline three-day food diary; week-8 adherence assessed using a researcher-administered 24-h dietary recall	↓ Body weight, WC, HC, BMI, WHR, waist/height ratio, neck circumference, BFP ↓ FSH, TT, free thyroxine 4
Soares et al., 2016 Brazil [57]	n = 22 Age: 18–35 years BMI: ≥ 25- < 39 kg/m^2^ PCOS definition: Rotterdam ESHRE-ASRM-sponsored criteria	Pre-post intervention	12 weeks	CRD: 500 kcal reduction from the total daily energy requirement, consisting of 60% carbohydrates (8% simple sugar), 15% proteins, and 25% total fats (7% saturated fat). Dietary fiber intake was 25 g	None	Twenty-four-hour dietary recalls were conducted before and after the intervention	↓ Body weight. BMI ↓ FSH, SHBG, TT ↑ Progesterone ↓ FG, FI, HOMA-IR, LDL-C ↑ QUICK
Swora-Cwynar et al., 2016, Poland [47]	n = 77 (I: 39; C:38) Age: 18–40 years BMI: ≥ 30 kg/m^2^ PCOS definition: NR	RCT	12 weeks	*CRD: Consisting of 50–55% carbohydrates, 20–25% proteins, and 25% total fats, with < 300 mg cholesterol	An isocaloric diet combined with metformin	Dietary intake reviewed every 14 days by a dietitian using interviews and food diaries	↓ Body weight, BMI, WC, BFP, BFM, LBM%
*Intermittent Fasting*							
Abu Salma et al., 2024 Jordan [23]	n = 86 (I: 57; C: 29) Age: 19–40 years BMI: > 25 kg/m^2^ PCOS definition: Rotterdam criteria	RCT	6 months	IF: 500 kcal reduction from the total daily energy requirement, consisting of 50% carbohydrates, 20% proteins, and 30% total fats. Fast for 18 h on non-consecutive days up to three times per week	CRD: 500 kcal reduction from the total daily energy requirement, consisting of 50% carbohydrates, 20% proteins, and 30% total fats	Food diary (two weekdays and one weekend day) collected during the initial phase; nutrient intake analyzed	IF: ↓ Body weight, BMI, BFM, BFP, FFM, VAT, MM CRD: ↓ Body weight, BMI, BFM, BFP, VAT
Cezlan and Sevim, 2024 Turkiye [27]	n = 33 (I_1_: 11; I_2_:11; C:11) Age: 18–45 years BMI: 25–35 kg/m^2^ PCOS definition: NR	RCT	8 weeks	IF: 16:8 intermittent fasting model, with the first meal generally scheduled at 11:00 and the second at 19:00 CRD: 500 kcal reduction from the total daily energy requirement, consisting of 45–60% carbohydrates, 15–20% proteins, and 25–30% total fats	Participants maintained their usual diet	Baseline three-day consecutive food records collected via detailed interview	CRD: ↓ Body weight, BMI, BMR, WC, HC, MUAC, neck circumference, BFP, BFM, VAT ↓ Arm fat (R/L), leg fat (L), trunk fat ↓ Leg muscle (R) IF: ↓ Body weight, BMI, BMR, WC, HC, MUAC, WHR, neck circumference ↓ Arm fat (R/L), leg fat (R/L), trunk fat ↓ FFM, BW Significant improvements were observed in the IF group compared to the CRD group: ↓ Body weight, BMI, BW, trunk fat
Feyzioglu et al., 2023 Turkiye [28]	n = 30 Age: 18–40 years BMI: 18–30 kg/m^2^ PCOS definition: Rotterdam criteria	Retrospective study	6 weeks	IF: Participants were free to eat and drink from 1 p.m. to 9 p.m. (8 h) and fast from 9 p.m. to 1 p.m. the next day (16 h)	None	NR	↓ BMI, WHR ↓ AMH, FSH, LH, E2, prolactin ↓ Calprotectin ↓ FI, FG, HOMA-IR, HbA1c, LDL-C, TG ↓ TT, free testosterone, FAI, DHEAS ↑ TSH, HDL-C ↑ SHBG ↓ Hyperandrogenism
Jiang et al., 2025 China [30]	n = 104 (I: 52; C: 52) Age: 22–45 years BMI: > 25 kg/m^2^ PCOS definition: Rotterdam criteria	RCT	2 months	IF: The 4-week cycle began with an intestinal conditioning and adaptation phase followed by phases of alternate-day fasting, with 600 kcal/day intake, and light fasting, with a 500 kcal/day energy deficit,, across different weekdays. Additionally, participants consumed 20 g of flaxseed powder twice daily before meals. In the second month, participants resumed a regular, unrestricted diet	Rice flour was a placebo substitute for staple food in combination with IF	NR	In both groups: ↓ BMI, WC, HC, WHR ↓ FSH, LH, E2, progesterone ↓ FG, FI, HOMA-IR, TC, TG, LDL-C ↑ HDL-C Significant improvements were observed in the IF group compared to the control group ↓ BMI ↓ FSH, LH, E2, progesterone ↓ FI, HOMA-IR, TC, TG, LDL-C
Li et al., 2021, China [32]	n = 15 Age: 18–40 years BMI: ≥ 24 kg/m^2^ PCOS definition: Rotterdam criteria	Quasi-experimental	6 weeks	IF: Instructed to eat freely from 8 am to 4 pm daily and to fast from 4 pm to 8 am the next day. Daily dietary calorie intake was required to be consistent with the baseline	None	Daily dietary intake logged using a food diary and the Boohee mobile application	↓ Body weight, BMI, BFM, BFP, VAT ↓ FI, HOMA-IR, AUCIns, AUCIns/AUCGlu ↑ SHBG ↓ TT, FAI ↓ CRP, ALT ↑ IGF-1
Zangeneh et al., 2015 Iran [43]	n = 40 (I: 20; C: 20) Age: 20–40 years BMI: NR PCOS definition: joint criteria of the ESHRE and the ASRM	Quasi-experimental	30 days	IF: Ramadan fasting	Participants maintained their usual diet	NR	↓ Cortisol ↓ Noradrenaline
*Others*							
Colonetti et al., 2025 Brazil [58]	n = 24 (I: 12; C: 12) Age: > 18 years BMI: NR PCOS definition: Rotterdam criteria	RCT	12 weeks	A low-carbohydrate diet consists of CHO values up to 40% of daily energy values, approximately 26% protein, and 34% lipids, and with strength exercise	Standard diet: Carbohydrate intake greater than 50% of the daily energy intake, approximately 20% protein, and 30% lipids, recommended with strength exercise	Monthly dietitian visits plus follow-up food consumption questionnaire	Significant improvements were observed in the control diet group compared to the low-carbohydrate group ↓ DHEA ↓ LH, FSH
Karamali et al., 2018 Iran [44]	n = 60 (I: 30; C: 30) Age: 18–40 years BMI: NR PCOS definition: Rotterdam criteria	RCT	8 weeks	Soy protein diet: 0.8 g/kg protein (35% animal, 35% soy, 30% vegetable); textured soy (20.3 g/day), rich in fiber and phytoestrogens; all on 1500 mg/day metformin	Similar macronutrient content, but 70% animal + 30% vegetable protein; no soy, all on 1500 mg/day metformin	Weekly telephone interviews plus three-day food records used for cross-verification	Significant improvements were observed in the soy protein diet group compared to the control group ↓ BMI ↓ FG, TT, free testosterone, FI, HOMA-IR, TG, VLDL-C, TC ↑ QUICKI ↓ MDA ↑ GSH, NO
Kataoka et al., 2019 Sweeden [51]	n = 72 (PCOS: 16; non-PCOS: 56) Age: 18–50 years BMI: ≥ 35 kg/m^2^ PCOS definition: NIH 1990	Prospective cohort study	12 months	VLCD: A structured weight loss program including 12-week VLED (450–800 kcal/day via 3–5 meal replacement products/day), followed by reintroduction of energy-restricted meals (1400–1600 kcal/day)	None	Monthly visits with a study dietitian	In the PCOS group: ↓ Body weight, BMI, HC, WC, WHR ↓ LDL-C ↓ DEXA total fat, DEXA total lean
Kazemi et al., 2018, Canada [59] 2020 [60]	n = 61 (I: 30; C:31) Age: 18–35 years BMI: NR PCOS definition: Androgen Excess and PCOS Society	RCT	16 weeks	Pulse-based diet: Pulse-rich diet was provided with two standard pulse meals (i.e., lunch and dinner) daily, containing approximately 90 g of split peas, 225 g of chickpeas, or beans, or 150 g of lentils (cooked weight). All women were enrolled in a low-impact aerobic training programme	TLC diet: Therapeutic Lifestyle Changes diet (lower fat, high fiber), which excludes pulses and is for energy and CHO. All women were enrolled in a low-impact aerobic training programme	Serial 24-h dietary recalls at baseline and monthly; daily food and exercise logs used in the follow-up study	Significant improvements were observed in the pulse-based diet group compared to the TLC diet group: ↓ TG, LDL-C, TC/HDL-C ↑ HDL-C
Mei et al., 2022 China [33]	n = 59 (I: 30; C:29) Age: 16–45 years BMI: ≥ 24 kg/m^2^ PCOS definition: Rotterdam criteria	RCT	12 weeks	Mediterranean diet + Low-carb (MED/LC): Max. 100 g CHO/day (< 20%), increased protein and fat intake. Participants were advised to consume whole grains as a staple food, a high intake of extra virgin olive oil and vegetables, a moderate intake of fish and other meat, dairy products, and a low intake of eggs	Low-fat diet: < 30% kcal from fat (< 40 g/day), SFA < 10%; increased the intake of cereals, vegetables, and fruits as appropriate	Weekly dietary guidance via WeChat plus daily dietary intake logged using the Boohee mobile application	MED/LC: ↓ Body weight, BMI, WC, WHR, BFP ↓ TT, LH, LH/FSH ↓ FG, FI, HOMA-IR, TG, TC, LDL-C ↑ QUIKI Low-fat diet: ↓ Body weight, BMI, WC, WHR, BFP ↓ TT, LH, LH/FSH ↓ FI, HOMA-IR, TG, TC, LDL-C ↑ QUIKI
Naji et al., 2024 Yemen [35]	n = 16 Age: 16–40 years BMI: NR PCOS definition: NR	Pre-post intervention	8 weeks	Specialized diet: Based on 15% protein, 25% fat, 60% low-GI carbs, including fiber	None	NR	↓ BMI ↓ Hair loss, acne ↑ Regular menstrual days
Ozdemir et al., 2025 Turkiye [36]	n = 30 (I:14; C:16) Age: 19–35 years BMI: > 25 kg/m^2^ PCOS definition: Rotterdam criteria	RCT	12 weeks	Energy-restricted Low-AGEs containing diet: 500–1000 kcal/day reduction, 20–25% fat, cooking methods restricted to boiling, steaming, marinating; avoidance of high-AGE industrial and roasted foods	Energy-restricted Standard-AGEs containing diet: Same energy restriction, no change in cooking practices	Interviews at weeks 6 and 12 plus baseline three-day food records	In the Low-AGEs group: ↓ Body weight, BMI, WHR, BFP, trunk fat%, visceral fat level ↓ FI, HOMA-IR, LDL-C, TNF-α ↑ QUICKI ↓ TT, FAI, AMH ↑ SHBG In the Standard-AGEs group: ↓ Body weight, BMI, BFP, trunk fat%, visceral fat level ↓ FI, HOMA-IR, TAS Significant improvements were observed in the low AGEs group compared to the control group ↓ FG
Papakonstantinou et al., 2016 Greece [52]	n = 40 Age: 27 ± 6 years BMI: 27 ± 6 kg/m^2^ PCOS definition: Rotterdam criteria	Randomized crossover trial	24 weeks	Six-meal isocaloric pattern: 40% carbohydrates, 25% protein, 35% fat. A six-meal pattern was followed for 12 weeks, and then participants switched to the three-meal pattern for another 12 weeks	Three-meal isocaloric pattern: 40% carbohydrates, 25% protein, 35% fat. A three-meal pattern was followed for 12 weeks, and then participants switched to the six-meal pattern for another 12 weeks	Detailed food diaries reviewed biweekly by dietitians; dietary adjustments made when necessary	In the six-meal pattern group: ↓ WC In the three-meal pattern group: ↓ WC, HC ↑ FI Significant improvements were observed in the six-meal pattern group compared to the control group: ↓ FI, Matsuda index
Phy et al., 2015 USA [61]	n = 24 Age: 18–45 years BMI: 25–45 kg/m^2^ PCOS definition: Rotterdam criteria	Prospective	8 weeks	Ad libitum low-starch/low-dairy diet: Participants were instructed to eat lean animal protein (meat and poultry), fish and shellfish, eggs, non-starchy vegetables, low-sugar fruits, avocado, olives, nuts and seeds, and oils, and were allowed up to 1 oz, of prepared or fresh, full-fat cheese per day	None	Three-day food records collected at weeks 1, 4, and 7	↓ Body weight, BMI, WC, WHR, BFP ↓ FG, FI, HOMA-IR, HbA1c, VLDL-C, HDL-C, TG ↓ TT, free testosterone ↑ 25-OH Vitamin D ↓ Hirsutism
Sanlı Ak et al., 2017 Turkiye [38]	n = 20 (I:10; C:10) Age: 25–35 years BMI: 25–35 kg/m^2^ PCOS definition: NR	Quasi-experimental	6 weeks	HPD: Consisting of 40% carbohydrates, 30% protein, and 30% fat with oral antidiabetics	Normal protein diet: It consists of 55% carbohydrates, 15% protein, and 30% fat along with oral antidiabetics	Three-day food records plus weekly dietary follow-up visits	Significant improvements were observed in the HPD group compared to the control group: ↓ Body weight, BMI, WC, HC, neck circumference, waist/height ratio, BFP, BFM, LBM, FFM, TBW, TBW%, VAT, BMR

↓ Significantly decreased; ↑ Significantly increased. NR: Not reported. AMH: Anti-Müllerian Hormone; APOA: Apolipoprotein A; ASRM: American Society of Reproductive Medicine; ALT: Alanine Aminotransferase; AST: Aspartate Aminotransferase; BFM: Body Fat Mass; BFP: Body Fat Percentage; BMI: Body Mass Index; BMR: Basal Metabolic Rate; BW: Body Water; C: Control; CRD: Calorie-Restricted Diet; CRP: C-Reactive Protein; DASH: Dietary Approaches to Stop Hypertension; DBP: Diastolic Blood Pressure; DEXA: Dual-Energy X-ray Absorptiometry; DHEAS: Dehydroepiandrosterone Sulfate; DHEA: Dehydroepiandrosterone; DPPH: 2,2′-Diphenyl-1-picrylhydrazyl; E2: Estradiol; ESHRE: European Society of Human Reproduction and Embryology; FAI: Free Androgen Index; FFM: Fat-Free Mass; FG: Fasting Glucose; FI: Fasting Insulin; FSH: Follicle-Stimulating Hormone; FT: Free Testosterone; HbA1c: Glycated Hemoglobin; HGLD: High Glycemic Load Diet; HDL: High-Density Lipoprotein; HC: Hip Circumference; HOMA-B: Homeostasis Model Assessment-estimated β-cell Function; HOMA-IR: Homeostatic Model Assessment of Insulin Resistance; Hs-CRP: High-Sensitivity C-Reactive Protein; HPD: High-Protein Diet; I: Intervention; IF: Intermittent Fasting; IGF-1: Insulin-like Growth Factor 1; KD: Ketogenic Diet; LBM: Lean Body Mass; LGID: Low-Glycemic Index Diet; LGLD: Low-Glycemic Load Diet; LH: Luteinizing Hormone; LMM: Lean Muscle Mass; MD: Mediterranean Diet; MDA: Malondialdehyde; MM: Muscle Mass; MUAC: Mid-Upper Arm Circumference; NIH: National Institutes of Health; NO: Nitric Oxide; Progesterone: Progesterone; QUICKI: Quantitative Insulin Sensitivity Check Index; SHBG: Sex Hormone-Binding Globulin; TAS: Total Antioxidant Status; TC: Total Cholesterol; TG: Triglycerides; TNF-α: Tumor Necrosis Factor-alpha; TSH: Thyroid-Stimulating Hormone; TT: Total Testosterone; VAT: Visceral Adipose Tissue; VLCD: Very-Low-Calorie Diet; VLCKD: Very-Low-Calorie Ketogenic Diet; WC: Waist Circumference; WHR: Waist-to-Hip Ratio; 25-OH Vitamin D: 25-Hydroxy Vitamin D

^*^In this study, the results of the group that received only the dietary intervention were evaluated

### Relationship between dietary ınterventions and hormones

Several studies reported increases in SHBG levels following dietary interventions [25, 26, 28, 29, 32, 36, 39, 40]. However, in women with PCOS who are living with overweight or obesity, a 12-week intervention involving a hypocaloric diet with a 500 kcal/day energy deficit resulted in decreased SHBG levels [57]. Among reproductive hormones, a significant reduction in prolactin levels was reported in two studies [28, 39]. In addition, significant decreases were observed in the levels of androstenedione [26], E2 [28, 30], TT [28, 32, 33, 36, 37, 44, 57, 61], FT [28, 34, 44, 50, 61], and FAI [26, 28, 29, 32, 36, 40]. Similarly, reductions in DHEAS [25, 28] and DHEA [58] levels were also reported. While several studies showed a decline in AMH levels [28, 29, 36], one study found a significant decrease only among participants with PCOS who showed improved menstrual cyclicity after a calorie-restricted diet [34].

Gonadotropins were also affected, with reductions in LH [25, 28, 30, 33, 58], FSH [28, 30, 37, 57, 58], and the LH/FSH ratio [33, 62]. Findings related to progesterone levels were inconsistent across studies. At the same time, an increase was reported in one study involving a calorie-restricted diet [57]; another study reported a decrease following a four-week IF model combined with the daily intake of 20 g of flaxseed powder (twice per day) [30].

In addition to reproductive hormones, significant changes were observed in other hormones related to metabolism, stress response, and energy balance. Findings on IGF-1 levels varied across studies: a decrease was reported following a KD [39], whereas increases were observed in studies involving IF [32] and CRD [49]. Increases in QUICKI scores, indicating improved insulin sensitivity, were reported in multiple studies [29, 33, 36, 44, 57]. Additionally, increased TSH [25] and decreased leptin levels [54] were observed. Ramadan fasting interventions showed significant reductions in cortisol and noradrenaline [43]. Furthermore, in a randomized crossover study comparing high and low glycemic load diets, a decrease in ghrelin levels was observed at the 240^th^ minute in the low glycemic load group, while glucagon levels increased at 0, 60, 120, and 180 min [55] (Table 6).

### Relationship between dietary ınterventions and metabolic outcomes

Dietary interventions were associated with significant changes in glycemic control, lipid profile, liver enzymes, inflammatory, and antioxidant markers in women with PCOS. Significant reductions in FG [28, 30, 33, 36, 39, 42, 44, 49, 53, 57, 61], FI [24, 28–30, 32–34, 36, 39, 40, 44, 49, 53, 57, 61], and HOMA-IR [24, 28–30, 32, 33, 36, 39, 40, 44, 50, 53, 57, 61] levels were reported in numerous studies, whereas a decrease in HOMA-B was reported in only one study [29]. A reduction in HbA1c levels was reported in only two studies [28, 61].

Regarding blood lipid parameters, significant reductions in TG [28, 30, 33, 42, 44, 45, 53, 59, 61] were consistently observed across studies. While TC showed conflicting results, an increase was reported in a study by Yang et al. [42], which investigated a KD in women with PCOS who have overweight or obesity, particularly among those without hyperuricaemia. In contrast, other studies demonstrated decreases in TC levels [30, 33, 44, 45, 50]. Similarly, HDL-C levels varied, with some studies indicating a reduction [39, 61] and others reporting an increase [28, 30, 45, 59]. A few studies found LDL-C levels to increase [42, 51, 59], but the majority reported significant reductions [28, 30, 33, 36, 45, 50, 57]. Additionally, decreases in VLDL-C concentrations were noted in two studies [44, 61].

Regarding liver enzymes, reductions in ALT were reported in both the IF study by Li et al. [32] and the KD study by Yang et al. [42], while a decrease in AST was observed only in the latter.

Findings related to inflammatory and oxidative stress markers showed variable results across studies. Changes in TNF-α levels varied depending on the type and duration of dietary intervention. While an increase in TNF-α was reported following a short-term (4-week) KD in the quasi-experimental study by Seven Avuk et al. [39], a significant reduction was observed in the randomized controlled trial conducted by Özdemir et al. [36], in which participants followed a low-AGEs energy-restricted diet for 12 weeks. High-sensitivity C-reactive protein (hs-CRP) levels were consistently reduced following dietary interventions in several studies [24, 32, 62]. Regarding oxidative stress parameters, reductions in MDA [29, 44] and increases in NO [29, 40, 44] were reported. Some studies also demonstrated improvements in antioxidant status, such as increased DPPH radical scavenging activity [26], glutathione peroxidase [46], and GSH [44]. However, a decrease in TAS was observed in one study [36], indicating that responses across antioxidant markers were not consistent.

In addition to metabolic improvements, some studies reported favorable changes in secondary clinical parameters. A significant reduction in DBP was observed in the LGID intervention by Shishehgar et al. [40], while an increase in serum 25-OH D levels was reported in the low-starch/low-dairy diet study by Phy et al. [61], suggesting potential ancillary benefits of dietary modification in women with PCOS (Table 6).

### Relationship between dietary ınterventions and reproductive and clinical symptoms

Ketogenic diet intervention in women with PCOS resulted in significant improvements in cycle implantation rate, clinical pregnancy rate, live birth rate, and ongoing pregnancy/live birth rate [53]. Similarly, in an LGID intervention conducted by Becker et al. among women with PCOS experiencing infertility, the pregnancy rate increased to 21.4% and oocyte retrieval also increased [54]. In another study where LGID was implemented for 24 weeks, a reduction in menstrual cycle length and increased menstrual frequency and regularity were observed [40]. Furthermore, a separate LGID trial involving women diagnosed with infertility and anovulation showed a higher frequency of ovulatory cycles in the intervention group compared to the control group [56]. In a study where a 1200-kcal energy deficit was applied, a decrease in the frequency of oligomenorrhea was reported in women with PCOS [48]. Dietary interventions were also found to be effective in improving dermatological symptoms. Several studies reported a reduction in acne severity [35, 40, 62] and a decrease in hirsutism levels [40, 48, 61, 62]. Only one study reported that IF led to a reduction in hyperandrogenic symptoms [28]. In contrast, another study found that an 8-week specialized diet reduced hair loss and increased the number of regular menstrual days [35] (Table 6).

## Discussion

The effects of different dietary models on PCOS have been the subject of many studies, and several systematic reviews and meta-analyses have been conducted. In this systematic review, we evaluated only studies involving dietary interventions and excluded multi-component interventions combining physical activity, pharmacological treatments, or dietary supplements. This approach was chosen to clearly demonstrate the independent effect of diet. PCOS is an endocrine-metabolic disorder with a lifelong course, heterogeneous clinical features, and an etiology that has not yet been fully elucidated. Neuroendocrine imbalances, genetic and epigenetic factors, environmental pollutants, gut microbiota dysbiosis, obesity, unhealthy dietary habits, physical inactivity, and other environmental influences all contribute to its development [63].

The relationship between IR and PCOS is explained by increased serine phosphorylation and decreased tyrosine phosphorylation of insulin receptors and insulin receptor substrate-1 (IRS1), leading to impaired downstream insulin signaling [64]. Insulin resistance plays an accelerating role in the development of obesity, and this interaction creates a vicious cycle among the risk factors for PCOS [63]. Therefore, diets aiming at weight loss have come to the forefront in the management of PCOS [65]. Reducing risk factors and implementing personalized nutritional interventions emerge as essential targets in treatment strategies [66, 67].

Our findings demonstrated that different dietary patterns led to significant reductions in anthropometric measurements in women with PCOS. These results are consistent with previous studies reporting that dietary interventions can improve PCOS symptoms and metabolic risk factors through weight loss [66–68]. Being overweight is known to increase susceptibility to PCOS, whereas weight loss positively affects insulin metabolism and ovulation rates [67, 69]. It should be noted that variability in baseline characteristics across studies, particularly age, BMI, and nutritional status, may partly explain inconsistencies in reported outcomes. Since obesity, insulin resistance, and metabolic status significantly influence endocrine and reproductive responses in women with PCOS, differences in participant profiles may have contributed to the heterogeneity observed across dietary interventions.

A recent network meta-analysis comparing various dietary patterns in women with PCOS reported that the LGID was the most effective diet in reducing BMI, while the DASH diet provided the greatest improvements in HOMA-IR, FI, and FG levels. Although no significant differences were observed in hormonal parameters, only TG levels showed greater improvements with the DASH and low-carbohydrate diets [14]. The LGI diet also resulted in significant reductions in blood lipid levels, though its effects on glycemic control were limited in some studies [66, 70]. Similarly, studies on the KD have reported improved lipid parameters and, in some cases, additional benefits in glycemic control. Meta-analyses evaluating the effects of the KD have demonstrated improvements in blood lipid levels (TG, TC, LDL-C), glycemic parameters (HOMA-IR, FI), and hormonal markers (LH, TT, and FSH) [71–73]. In addition, Wang et al. reported that high-protein diets improved HOMA-IR, lipid profiles, and reproductive hormones [74].

Several studies have also demonstrated that dietary interventions led to reductions in androgen-related outcomes, including SHBG, TT, FT, FAI, and overall androgen levels. In particular, the DASH diet has shown beneficial effects on SHBG and anti-Müllerian hormone (AMH) [26, 29]. Short-term applications of the KD have demonstrated notable decreases in LH, TT, and free testosterone [66, 67]. However, long-term animal studies have indicated that the KD may increase hepatic steatosis and glucose intolerance, raising concerns about its long-term feasibility [75]. A meta-analysis revealed that, in individuals with PCOS, the most effective strategy for improving TT, FSH, and LH levels was the combination of exercise, diet, and pharmacological treatment. In the same study, no effects of these interventions were observed on progesterone levels [76].

The impact of nutrition on reproductive outcomes is also important. While balanced and adequate nutrition has been shown to increase the likelihood of conception and live birth rates in healthy women, it is also plausible that, in women with PCOS, dietary improvements may enhance disease-related parameters while increasing the chance of conception [77]. In a study conducted by Haase et al., an inverse relationship between BMI and the probability of conception was reported [78]. Becker et al. found that in women with PCOS who are living with obesity and experiencing infertility following a LGID, pregnancy rates increased to 21.4% along with an improvement in oocyte count. Long-term LGID interventions have also been shown to improve menstrual cycle regularity and ovulatory cycle frequency [67]. Similarly, Palafox-Gomez et al. demonstrated that the KD improved implantation, clinical pregnancy, live birth, and ongoing pregnancy rates [53]. However, in lifestyle interventions (diet + exercise) conducted among pregnant women with and without PCOS who are overweight or have obesity, no significant differences were observed in perinatal outcomes [68]. This suggests that interventions may be more effective prior to conception. Moreover, Ruiz-Gonzalez et al. emphasized that the combined application of diet, exercise, and pharmacological treatments increased ovulation rates, an effect particularly associated with reductions in BMI [76].

Taken together, these findings indicate that different dietary approaches may exert differential effects across outcome domains in women with PCOS. Ketogenic and LGID appeared to demonstrate more consistent improvements in anthropometric measures and certain reproductive outcomes, whereas DASH and CRD interventions were more frequently associated with favorable changes in metabolic risk markers, including glycemic parameters and lipid profiles. Intermittent fasting and other dietary models yielded more variable findings, which seemed to depend largely on intervention duration, baseline participant characteristics, and the selected outcome. Importantly, these observations are based on a narrative synthesis of heterogeneous studies, and no direct head-to-head comparisons between dietary approaches were performed. The diversity of dietary approaches and outcome measures across studies highlights the complexity of synthesizing findings within a single quantitative framework.

This systematic review has certain limitations. Variations in dietary interventions, diagnostic criteria for PCOS, and outcome assessment methods made it difficult to compare studies. Sample sizes also varied considerably, and some outcomes were assessed in only a limited number of studies. In addition, incomplete reporting of baseline characteristics, such as age, BMI, and nutritional status, in several included studies may have influenced the interpretation of anthropometric, endocrine, metabolic, and reproductive outcomes. Since most interventions were of relatively short duration, there is insufficient evidence regarding the long-term effects of dietary modifications in women with PCOS.

Nevertheless, this review also presents important strengths. Only studies involving dietary interventions were included, allowing the independent effects of diet to be evaluated without the influence of other lifestyle factors. Furthermore, the review synthesizes a wide body of literature from the last decade, encompassing anthropometric, metabolic, hormonal, and clinical outcomes, and applies JBI appraisal tools for methodological quality assessment. Its adherence to PRISMA guidelines and prior PROSPERO registration also enhances the finding’s transparency and reliability.

## Conclusion

In conclusion, the findings of this systematic review, together with the existing literature, indicate that different dietary patterns can exert beneficial effects on metabolic, hormonal, anthropometric, and reproductive parameters in women with PCOS. However, given the persisting uncertainties regarding the long-term sustainability and effectiveness of dietary interventions, personalized and sustainable nutritional approaches should be prioritized in PCOS management; tailored to individuals’ baseline BMI, IR status, lifestyle, and dietary habits rather than recommending a single ideal dietary model. Furthermore, there is a clear need for large-scale, long-term randomized controlled trials evaluating the effects of dietary interventions alone. In addition, future research may benefit from focused meta-analyses restricted to homogeneous dietary models and standardized outcome measures to provide more precise quantitative estimates within clearly defined clinical subgroups. The current body of literature also highlights the lack of an internationally recognized clinical guideline on PCOS and nutrition.

## Supplementary Information

Below is the link to the electronic supplementary material.

Supplementary Material 1

Supplementary Material 2

## Data Availability

The datasets generated and/or analyzed during the current study are available from the corresponding author on reasonable request.
