# Supplementary material for: Nutrition interventions in women with polycystic ovary syndrome: a systematic review
Source: Eur J Nutr. 2026 Jun 29;65(5):176. doi: 10.1007/s00394-026-04030-7 (PMC13314694; doi:10.1007/s00394-026-04030-7)
Supplement: Supplementary file 2 — Supplementary Material 2 [file 394_2026_4030_MOESM2_ESM.docx]

**Databases and Search Results**

| **Databases** | **Results** |
| --- | --- |
| **Pubmed** |  |
| ("polycystic ovary syndrome"[Title/Abstract] OR "PCOS"[Title/Abstract] OR "polycystic ovarian syndrome"[Title/Abstract] OR "polycystic ovary disease"[Title/Abstract] OR "ovarian cysts"[Title/Abstract] OR "stein-leventhal syndrome"[Title/Abstract] OR "stein leventhal syndrome"[Title/Abstract]) AND ("diet*"[Title/Abstract] OR "nutrition*"[Title/Abstract] | 114 |
| **Cochrane Library** |  |
| ("polycystic ovary syndrome" OR "PCOS" OR "polycystic ovarian syndrome" OR "polycystic ovary disease" OR "ovarian cysts" OR "stein-leventhal syndrome" OR "stein leventhal syndrome") AND ("diet" OR "nutrition") | 528 |
| **EBSCO** |  |
| (TI (("polycystic ovary syndrome" OR "PCOS" OR "polycystic ovarian syndrome" OR "polycystic ovary disease" OR "ovarian cysts" OR "stein-leventhal syndrome" OR "stein leventhal syndrome")  AND ("diet*" OR "nutrition*")))  AND  (AB (("polycystic ovary syndrome" OR "PCOS" OR "polycystic ovarian syndrome" OR "polycystic ovary disease" OR "ovarian cysts" OR "stein-leventhal syndrome" OR "stein leventhal syndrome")  AND ("diet*" OR "nutrition*")))  NOT (TI ("review of literature" OR "literature review" OR "meta-analysis" OR "systematic review")) | 777 |
| **Science Direct** |  |
| ("polycystic ovary syndrome" OR "PCOS" OR "polycystic ovarian syndrome" OR "polycystic ovary disease" OR "ovarian cysts" OR "stein-leventhal syndrome" OR "stein leventhal syndrome") AND ("diet" OR "nutrition") | 173 |
| **Web of Science** |  |
| TS=(("polycystic ovary syndrome" OR "PCOS" OR "polycystic ovarian syndrome" OR "polycystic ovary disease" OR "ovarian cysts" OR "stein-leventhal syndrome" OR "stein leventhal syndrome") AND ("diet*" OR "nutrition*")) | 1187 |
| **Google Scholar** |  |
| (intitle:"polycystic ovary syndrome" OR intitle:"PCOS" OR intitle:"polycystic ovarian syndrome" OR intitle:"polycystic ovary disease" OR intitle:"ovarian cysts" OR intitle:"stein-leventhal syndrome" OR intitle:"stein leventhal syndrome") AND (intitle:"diet" OR intitle:"nutrition") | 400 |
| **DergiPark** |  |
| ((title:("polycystic ovary syndrome" OR "PCOS" OR "polycystic ovarian syndrome" OR "polycystic ovary disease" OR "ovarian cysts" OR "stein-leventhal syndrome" OR "stein leventhal syndrome") AND title:("diet" OR "nutrition"))) OR ((abstract:("polycystic ovary syndrome" OR "PCOS" OR "polycystic ovarian syndrome" OR "polycystic ovary disease" OR "ovarian cysts" OR "stein-leventhal syndrome" OR "stein leventhal syndrome") AND abstract:("diet" OR "nutrition"))) | 18 |
| **National Thesis Center** |  |
| polycystic ovary syndrome | 39 |
| *Filters for year, article type, language, and species were applied in the databases where such options were available | |
